# Combined Use of Magnetization Transfer Ratio and T2-Mapping to Evaluate Extraocular Muscle Pathophysiology in Myasthenia Gravis with Ophthalmoparesis

**DOI:** 10.7150/ijms.104542

**Published:** 2024-10-28

**Authors:** Qin Zhou, Xiaoxiao Zhao, Mengzhu Wang, Yingkai Li, Zhiyun Yang, Weibin Liu, Pei Chen

**Affiliations:** 1 Department of Radiology, The First Affiliated Hospital, Sun Yat-sen University, Guangzhou, China.; 2 MR Scientific Marketing, Siemens Healthineers Ltd. Guangzhou, China.; 3 Neuromuscular Division, Department of Neurology, Duke University Medical Center, Durham, United States.; 4 Department of Neurology, The First Affiliated Hospital, Sun Yat-sen University, Guangzhou, China.; 5 Guangdong Provincial Key Laboratory of Diagnosis and Treatment of Major Neurological Diseases, Guangzhou, China.; 6 National Key Clinical Department and Key Discipline of Neurology, Guangzhou, China.

**Keywords:** Myasthenia gravis, extraocular muscle, magnetization transfer imaging, T2-mapping, magnetization transfer ratio, ophthalmoparesis

## Abstract

**Background** Myasthenia gravis (MG) is an autoimmune neuromuscular disorder that most frequently affects the extraocular muscles (EOMs), which causes symptoms such as ptosis and restricted eye movement. The EOMs in MG patients are representative of autoimmune inflammatory changes in muscle tissue. Currently, there is no reliable, and sensitive imaging technique for monitoring EOM changes to assist in the evaluation of underlying pathological changes.

**Methods** This study included MG patients treated between March and November 2022 at the First Affiliated Hospital of Sun Yat-sen University. Healthy controls (matched by age and sex) were included. Participants underwent 3.0 T MRI with magnetization transfer imaging (MTI) and T2-mapping to measure the magnetization transfer ratio (MTR) and T2-mapping values in the superior, inferior, medial, and lateral rectus muscles. Comparisons were made between MG patients and healthy controls, and between MG subgroups with and without ophthalmoparesis.

**Results** The MTR and T2-mapping values successfully reflected EOM fibrosis and inflammatory edema in MG patients. MG patients showed significantly higher MTR and T2-mapping values in the EOMs compared with healthy controls. MG patients with ophthalmoparesis exhibited a lower MTR but higher T2-mapping value compared with those without ophthalmoparesis. Combined MTR and T2-mapping values effectively distinguished between MG patients and healthy controls, and between different severities of EOM involvement, with a superior diagnostic accuracy compared with each parameter alone.

**Conclusion** The combination of MTI and T2-mapping MRI techniques can provide key insight into the pathological changes in EOMs in MG patients. This approach enhances early diagnosis and treatment planning, and therefore may improve clinical outcomes.

## Background

Myasthenia gravis (MG) is a typical neuroimmunological disorder caused by autoantibodies, which disrupt neurotransmitter signaling at the neuromuscular junction and cause abnormal skeletal muscle fatigue 1, 2. The extraocular muscles are the most affected skeletal muscles 3. Although most MG patients do not exhibit symptoms, persistent eyelid drooping and fixed or significantly restricted eye movement are frequently observed clinically, which can severely impact upon patients' daily life and appearance. MG patients often have a long medical history, and their extraocular muscles are significantly atrophied (suggestive of severe inflammation), particularly early in the disease course, which affects their function 4. Currently, there are limited techniques to quantitatively assess the extent of extraocular muscle involvement in MG patients in the early stages, and it is only possible to judge the extent of involvement based on the degree of eye movement limitation at that time 5. Furthermore, the extraocular muscle symptoms have received minimal clinical attention, which has limited the development of effective treatment strategies.

T2 mapping (i.e., the T2 relaxation time map) is a quantitative analysis technique that can measure the T2 value of tissues, and reflects changes in the extracellular fluid and collagen content 6. T2 mapping is commonly used as a quantitative measure of water content in muscle tissue for assessment of inflammatory edema 7. This MRI sequence is also often applied for clinical assessment of a range of diseases including Graves' ophthalmopathy (GO) 7, 8. Magnetization transfer imaging (MTI) primarily describes the physical process of magnetization transfer between free hydrogen nuclei and those bound to macromolecules in tissues, and indirectly reflects the concentration of macromolecules 9, 10. MTI has been recently used to assess the degree of pancreatic fibrosis before surgery 11, differentiate between fibrotic and inflammatory bowel strictures in Crohn's disease 12, and evaluate fibrosis following radiotherapy for rectal cancer 13, among other conditions 14, 15.

To date, no studies have used MTI and T2 imaging to assess the inflammatory state of the extraocular muscles in MG patients. In the present study, we used MTI and T2 mapping sequences to evaluate the extraocular muscles of MG patients for assessment of pathophysiological changes in the extraocular muscles, and we provide important information for clinical diagnosis and treatment.

## Materials and Methods

### Subjects

This was a prospective study of patients who were clinically diagnosed with MG at the First Affiliated Hospital of Sun Yat-sen University from March 2022 to November 2022. Inclusion criteria were (1) confirmed MG and (2) completed orbital MRI examinations that included coronal MTI and T2-mapping sequences. Exclusion criteria were (1) poor quality MRI images that did not meet the requirements for subsequent analysis, (2) concomitant hyperthyroidism, or (3) presence of other orbital or extraocular muscle disorders. Healthy controls were selected from another clinical study and underwent orbital MRI that included coronal MTI and T2-mapping sequences. The healthy controls included in the present study were matched by age and sex. Ethics approval for this study was obtained from the research ethics committee of the First Affiliated Hospital of Sun Yat-sen University (No. 2022-666). Informed consent was obtained from all participants. The authors affirm that human research participants provided informed consent to publish the images.

### MG patient subgroup definitions

MG patients were classified into the MG-O (with ophthalmoparesis) and MG-N (without ophthalmoparesis) groups. Ophthalmoparesis was defined as a limitation of eye movement in any direction at the time of the study, without consideration of ptosis 16.

### MRI protocol

All subjects underwent MRI in a 3.0-T scanner (Magnetom PRISMA; Siemens Healthineers, Erlangen, Germany) with a 64-channel transmit-receive head-coil (INVIVO, Gainesville, FL, USA). Patients were instructed to lie supine and close their eyes to reduce eye movement. The MTI was performed using two coronal three-dimensional gradient echo sequences with or without an off-resonance pre-pulse. The scanning parameters were: repetition time = 34 ms, echo time = 6 ms, field of view = 220×165 mm², matrix = 256×256, bandwidth = 160 Hz/Px, 16 slices, slice thickness = 3 mm, voxel size = 0.9×0.9×3.0 mm^3^, flip angle = 10°, and acquisition time = 1:43 min. T2-mapping data were obtained using a T2 multi-spin-echo sequence. The acquisition parameters of the T2 sequences as following: repetition time=2000 ms, echo time=13.8, 27.6, 41.4, 55.2, 69.0ms, matrix 384×384, flip angle = 180°, bandwidth = 228 Hz/Px, 18 slices, slice thickness 3.0 mm and acquisition time = 4:54 min.

### Image analysis

**Figure 1:**
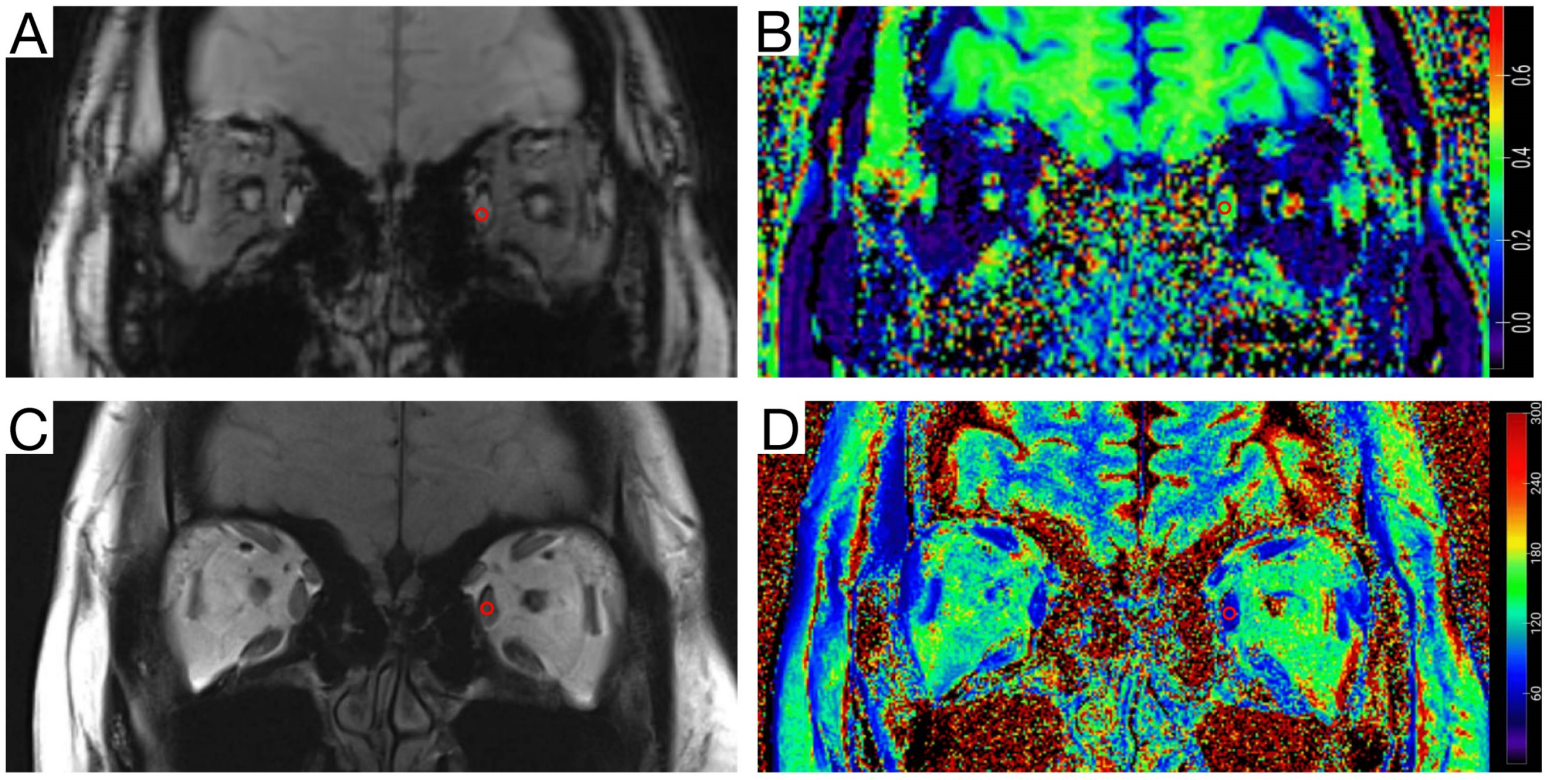
Measurement diagram of the magnetization transfer ratio (MTR) and the T2-mapping value. A circular region of interest (ROI) (area of 5-10 mm^2^) was delineated on the coronal orbital T2 anatomical mapping image with the largest cross-sectional area of the ocular muscles of myasthenia gravis (MG) patients and healthy volunteers. The ROI was then copied to the T2-mapping image, and the corresponding T2 signal values were recorded. On the same level of the Magnetization transfer off (MT-off) map, the same ROI (area of 5-10 mm^2^) was delineated, the ROI was copied to the MTR image, and the corresponding MTR value was recorded. **A**, MT-off image. **B**, MTR image. **C**, T2-anatomical mapping image. **D**, T2-mapping image.

The magnetization transfer ratio (MTR) images were generated using Matlab code (R2021a, Mathworks, MA, USA). The T2-mapping values were carried out using syngo.via (VB20A_HF91) at Siemens workstation (Siemens Healthineers, Erlangen, Germany). MTR was calculated as MTR = 1 - (MT1/MT0) 12, 17, where MT0 is the local signal intensity of the gradient echo sequence without the magnetization transfer saturation pulse, and MT1 is the local signal intensity of the gradient echo sequence with the magnetization transfer saturation pulse. On the MT-off images and T2 anatomical mapping images, a roughly circular region of interest (ROI) was manually drawn on the largest coronal section of the superior, medial, inferior, and lateral rectus muscles. Then the ROI were copy to the MTR image and T2-mapping image (Figure 1). The MTR and T2-mapping values for the left and right eyes were measured and recorded separately. Finally, the mean of MTR and T2-mapping values of the bilateral four rectus muscles (total eight rectus muscles) were used for statistical analysis. The quantitative parameters were measured by two neuroradiologists with more than seven years of experience in head and neck imaging diagnosis, who were blinded to the clinical data. The second radiologist reperformed the measurements after 1 month to assess inter-observer and intra-observer consistency. The first measurement by the second radiologist was used for the final statistical analysis.

### Statistical analysis

Statistical analyses were performed using statistical software (SPSS v23.0, IBM Corp., Armonk, NY, USA; Prism v9.0, GraphPad software, Boston, MA, USA). The Kolmogorov-Smirnov test was used to assess the normality of quantitative data. Data following a normal distribution are expressed as mean±standard deviation (SD), and independent sample t-tests were used to compare the MTR and T2-mapping values between the two groups. Non-normally distributed data are expressed as median (Q1, Q3), and the Mann-Whitney U test was used to compare age between the two groups. Fisher's test was used to compare differences in categorical data between groups. A binary logistic regression model was used to combine the MTR and T2-mapping values. Receiver operating characteristic (ROC) curves were used to evaluate the predictive ability of the MRI quantitative parameters MTR, T2-mapping values, and their combination for extraocular muscle inflammation or fibrosis, while the DeLong method was used to compare the area under the curve (AUC) 18. A *p*-value <0.05 was considered statistically significant.

## Results

### Clinical data of enrolled patients

We enrolled 32 MG patients and 22 gender- and age-matched healthy controls (see Table 1 for age and gender data). The detailed clinical information of the enrolled patients and comparisons between the different MG subgroups are shown in Table 2.

### MG patients showed significantly higher means of MTR and T2-mapping values in the extraocular muscles compared with healthy controls

The mean MTR value of the extraocular muscles was significantly higher in MG patients compared with healthy controls (Table 2). Similarly, the mean T2-mapping value in MG patients was significantly higher than healthy controls (Table 2).

### Patients with ophthalmoparesis had a significantly lower mean MTR, but a higher mean T2-mapping value, than patients without ophthalmoparesis

**Figure 2:**
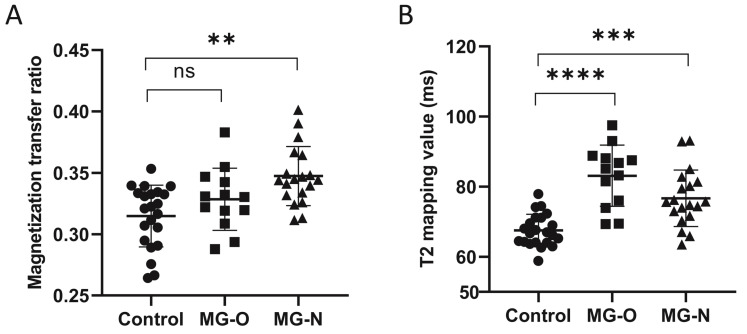
Comparison of the magnetization transfer ratio (MTR) **(A)** and the T2-mapping values** (B)** in healthy controls, myasthenia gravis (MG) patients with ophthalmoparesis (MG-O group), and MG patients without ophthalmoparesis (MG-N group). ns, not significant. **, *p* < 0.01; ***, *p* < 0.001; ****, *p* < 0.0001.

There is currently no clinical standard for assessing the severity of extraocular muscle involvement in MG patients. In the present study, we classified patients into those with (MG-O) and without (MG-N) ophthalmoparesis based on the extent of eye movement limitation. Patients with ophthalmoparesis are considered to have more severe extraocular muscle involvement. Of the 32 patients in the present study, 13 had ophthalmoparesis (MG-O) and 19 did not (MG-N). From the healthy controls, MG-N patients, and MG-O patients, the MG-N group had the highest average MTR (Fig. 2a), while the MG-O group had the highest average T2-mapping value (Fig. 2b).

### Combined MTR and T2-mapping values significantly differentiate between normal and MG-affected extraocular muscles

**Figure 3:**
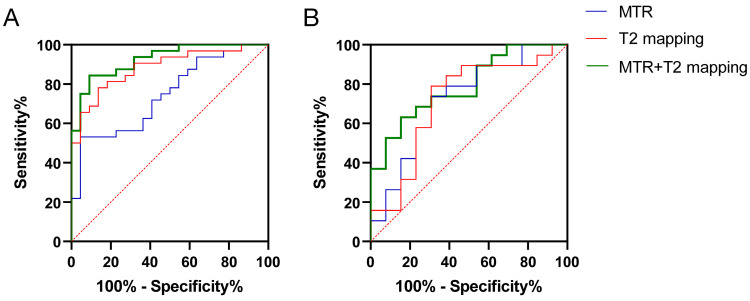
Receiver operating characteristic (ROC) curve analysis of MRI quantitative parameters (magnetization transfer ratio [MTR] and the T2-mapping value [ms]) for diagnosing and differentiating the severity of inflammation in the extraocular muscles of myasthenia gravis (MG) patients.** (A)** Diagnostic efficacy of combined MRI quantitative parameters (MTR and the T2-mapping value) in the extraocular muscles of MG patients. **(B)** Efficacy of combined MRI quantitative parameters (MTR and the T2-mapping value) in differentiating the severity of inflammation in the extraocular muscles of MG patients with ophthalmoparesis (MG-O) and without ophthalmoparesis (MG-N) (MG-O *vs.* MG-N groups). Blue line, MTR. Red line, T2-mapping value. Green line, combined MTR + T2-mapping value.

In Fig. 3A, ROC curve analysis showed that using an MTR of 0.3396 as a threshold to predict whether MG patients had extraocular muscle involvement resulted in an AUC (95% confidence interval (CI)) of 0.7443 (0.6138-0.8748; *p* = 0.0025), with a sensitivity of 53.13%, specificity of 95.45%, and accuracy of 68.52%. Using a T2-mapping value of 72.65 ms as a threshold, the AUC (95% CI) was 0.8835 (0.7954-0.9716; *p* < 0.001), with a sensitivity of 78.13%, specificity of 86.36%, and accuracy of 81.48%. There was no difference in AUC between the MTR and the T2-mapping value for predicting MG extraocular muscle involvement (Z = 1.732, *p* = 0.0832). The combined use of MTR and the T2-mapping values yielded an AUC (95% CI) of 0.9261 (0.8600-0.9923; *p* < 0.001), with a sensitivity of 84.38%, specificity of 90.91%, and accuracy of 87.04%. The combined predictive power of the MTR and the T2-mapping values was superior to MTR alone (Z = 2.436, *p* = 0.0149), but not significantly different from the T2-mapping value alone (Z = 0.7579, *p* = 0.4485).

### Combined MTR and T2-mapping values can differentiate the severity of extraocular muscle involvement in MG patients

In Fig. 3B, ROC curve analysis showed that using an MTR of 0.3330 as a threshold to predict severe extraocular muscle involvement in MG patients resulted in an AUC (95% CI) of 0.7206 (0.5341-0.9072; *p* = 0.0365), with a sensitivity of 73.68%, specificity of 69.23%, and accuracy of 71.88%. Using a T2-mapping value of 81.45 ms as a threshold, the AUC (95% CI) was 0.7126 (0.5181-0.9070; *p* = 0.0440), with a sensitivity of 78.95%, specificity of 69.23%, and accuracy of 71.88%. The difference in AUC between the MTR and the T2-mapping value for predicting severe extraocular muscle involvement in MG patients was not significant (Z = 0.0582, *p* = 0.9536). The combined use of MTR and the T2-mapping value yielded an AUC (95% CI) of 0.7895 (0.6338-0.9451; *p* = 0.0061), with a sensitivity of 63.16%, specificity of 84.62%, and accuracy of 71.88%. The combined predictive power of the MTR and the T2-mapping values was similar to MTR alone (Z = 0.5557, *p* = 0.5784) and the T2-mapping value alone (Z = 0.6051, *p* = 0.5451).

## Discussion

We previously described the utility of three-dimension time-of-flight magnetic resonance angiography sequences for measuring the extraocular muscle volume in MG patients 16. In the present study, we further used MTI and T2-mapping sequences and found that the MTR and the T2-mapping value of the extraocular muscles were significantly higher in MG patients compared with age-matched healthy controls. However, within the MG patient group, patients with ophthalmoparesis had lower MTR values but higher T2-mapping values compared with those without ophthalmoparesis.

Chronic inflammation often leads to fibrosis in muscle tissue, which results in an increase in connective tissue and large molecules 19, thereby elevating the MTR of muscle tissue 15, 20, 21. Thus, the MTR reflects the degree of tissue fibrosis. The inflammatory response caused by MG autoantibodies can primarily affect the extraocular muscles, which leads to related symptoms. Because MG is not curable and patients always have high levels of autoantibodies, their extraocular muscle tissues may remain in an extended state of chronic inflammation 4, 22. In turn, this may cause significant fibrosis, as reflected by an elevated MTR, in the extraocular muscles of MG patients, as shown in the present study.

T2-mapping values reflect the water content in muscle tissue 23. In GO, T2-mapping values are often used to quantify the degree of inflammatory edema in the extraocular muscles during the active phase of the disease 7, 8. Therefore, increased T2-mapping values could indicate acute inflammation in muscle tissue. Clinically, MG patients often experience relapses, and muscle weakness can signify worsening or recurrence of the condition. During the recurrence times, acute inflammation is occurring in the muscle tissue, which causes significantly higher T2-mapping values, especially in patients with ophthalmoparesis.

In the present study, ROC curve analysis of combined MTR and T2-mapping values showed significant differences between the extraocular muscles of MG patients and healthy controls, and using both measures together could distinguish between affected and normal extraocular muscles. However, in clinical practice, using MRI parameters of the extraocular muscles to diagnose MG may not be necessary, and we plan to explore new applications for these MRI sequences. For example, in studies of extraocular muscles in GO 8, combining MTR and T2-mapping values was helpful for assessing inflammatory activity. In MG patients, relying solely on clinical symptoms of extraocular muscle involvement to determine the degree of inflammation has notable limitations. The sensitivity of patients to extraocular muscle symptoms may vary. Some patients do not notice a significant impact on their lives from extraocular muscle symptoms, particularly if they lack diplopia or severe ptosis, and therefore may not prioritize these symptoms during medical visits. Additionally, neurologists may not always focus on extraocular muscle symptoms or link them to chronic inflammation. Given the relatively objective nature of MRI results, MRI may be a valuable complementary tool for evaluating inflammation in extraocular muscles.

Note that we used the range of eye movement as a clinical standard for assessing extraocular muscle involvement, with limited eye movement (i.e., ophthalmoparesis) considered an indicator of severe inflammation. Based on this standard, ROC curve analysis of combined MTR and T2-mapping values could differentiate between clinically different severities of extraocular muscle involvement. However, we observed some inconsistencies when we used the mean MTR and T2-mapping values to differentiate the degree of inflammatory response in the extraocular muscles of MG patients. For instance, MTR values were lower in patients with severe inflammation and ophthalmoparesis compared with healthy controls, despite the overall higher MTR values in MG patients. This may be caused by the significant increase in water content in the extraocular muscles during acute inflammation (i.e., higher T2-mapping values), which could lower the MTR values, as per the principles of MTR measurement 24, 25.

We previously reported that the extraocular muscle volume in MG-O patients was lower than that in MG-N patients, and that this volume was negatively correlated with disease duration in MG-N patients 16. These findings indirectly suggest that MG patients with ophthalmoparesis may be in a chronic inflammatory state, which leads to muscle fibrosis. In the present study, we further examined the relationship between MTR values and disease duration. To exclude the effects of edema from acute inflammation, we examined the correlations between MTR values and disease duration in MG-N patients. However, we found no significant correlation, which suggests that with long-term appropriate treatment, patients may effectively control muscle tissue inflammation and avoid fibrosis.

The significance of this study lies in its clinical application—a lower MTR and a higher T2-mapping value in an MG patient indicates severe inflammation in the extraocular muscles. Regardless of ophthalmoparesis, prompt and aggressive treatment may be appropriate^26^. This reflects the potential clinical significance of these MRI measurements. Moreover, because corticosteroids and immunosuppressants often have slow effects in treating MG, treatment adjustments may be delayed, which leads to suboptimal outcomes and increased side effects from prolonged medication use. MRI analysis of the extraocular muscles in MG patients can contribute to the early assessment of whether treatment is effectively controlling inflammation. Nevertheless, this study has several limitations, which include a small sample size, lack of more objective clinical standards for assessing extraocular muscle involvement, and the absence of detailed clinical evaluation standards that could allow different eyes of the same patient to be studied separately (as previously reported in GO 8), which would make it easier to collect sufficient clinical samples and conduct more precise studies.

In conclusion, analysis of the extraocular muscles in MG patients using special MRI sequences identified potential markers for assessing the degree of inflammatory extent in these muscles. These findings provide a reference for guiding precise treatment.

## Figures and Tables

**Table 1:** Comparison of the mean magnetization transfer ratio (MTR) and T2-mapping values between myasthenia gravis (MG) patients and healthy controls

	Ctrl (n=22)	MG (n=32)	T or Z value	P value
Age, years, mean±SD	43.91±22.04 (15-79)	44.97±18.11 (16-77)	0.1933	0.8474
Sex (male:female)	4:18	8:24		0.7416*
MTR, mean±SD	0.3147±0.02519	0.3397±0.02595	3.516	0.0009
T2-mapping value (ms), mean±SD	67.53±4.591	79.30±8.797	5.749	<0.0001

*, Fisher's test.

**Table 2:** Comparison of the mean magnetization transfer ratio (MTR) and T2-mapping values, and clinical characteristics, between myasthenia gravis (MG) patients with ophthalmoparesis (MG-O) and without ophthalmoparesis (MG-N)

		MG-O (n=13)	MG-N (n=19)	T or Z value	P value
MTR, mean		0.3285±0.02533	0.3473±0.02408	2.131	0.0414
T2 mapping value, mean		83.12±8.732	76. 69±8.043	2.147	0.0400
Age		49.54±17.17 (18-77)	41.84±18.51 (16-71)	1.189	0.2438
Sex (male:female)		7:6	1:18		
Antibody types, AChR: MuSK, n		12:1	16:3		
Duration, months, median, (IQR)		32.00 (7.55, 79.84)	25.10 (11.77, 61.30)		0.5962
Drug therapy, n					
	Pyridostigmine	13	19		
	Corticosteroid	11	17		
	Immunosuppressants	9	13		
Highest dose of corticosteroid, as prednisone, milligram, mean±SD		25.39±21.55	19.21±15.66	0.9402	0.3546
Kinds of immunosuppressant used, n					
	One	5	9		
	More than one	4	4		
MG subtypes, n					
	OMG	1	0		
	Early- onset GMG	3	12		
	Late-onset GMG	8	6		
	MG with thymoma	1	1		
MGFA type, n					
	I	2	0		
	II	3	7		
	III	6	11		
	IV	2	1		
	V	0	0		
Thymectomy status, n					
	Without thymectomy	7	17		
	Thymectomy	6	2		
Neurophysiology, n					
	RNS+	6	6		
	RNS- and SFEMG+	0	1		
	All negative	0	0		
	Unknown	7	12		

MG-N, MG patients without ophthalmoparesis; MG-O, MG patients with ophthalmoparesis; SD, standard deviation; AChR, acetylcholine receptor; MuSK, muscle-specific kinase; IQR, interquartile range; OMG, ocular MG; GMG, generalized MG; MGFA, myasthenia gravis foundation of America; RNS, repetitive nerve stimulation; SFEMG, single fiber electromyogram.

## Abbreviations

EOM: extraocular muscle
MRI: Magnetic resonance imaging
MTI: magnetization transfer imaging
MTR: magnetization transfer ratio
ROC: receiver operating characteristic
ROI: region of interest
GO: Graves' ophthalmopathy
MT: magnetization transfer
MG: myasthenia gravis
AChEI: acetylcholinesterase inhibitors
MuSK: muscle-specific kinase
MG-O: myasthenia gravis patients with ophthalmoparesis
MG-N: myasthenia gravis patients without ophthalmoparesis
SD: standard deviation
IQR: interquartile range
CI: confidence interval
AChR: acetylcholine receptor
MuSK: muscle-specific kinase
IQR: interquartile range
OMG: ocular MG
GMG: generalized MG
MGFA: myasthenia gravis foundation of America
RNS: repetitive nerve stimulation
SFEMG: single fiber electromyogram
